# Refining murine heterotopic heart transplantation: A model to study ischemia and reperfusion injury in donation after circulatory death hearts

**DOI:** 10.1002/ame2.12176

**Published:** 2021-07-07

**Authors:** Mohammed Quader, Renee Cholyway, Niluka Wickramaratne, Oluwatoyin Akande, Martin Mangino, Eleonora Mezzaroma, Adolfo G. Mauro, Qun Chen, Alexander Kantlis, Stefano Toldo

**Affiliations:** ^1^ Division of Cardiothoracic Surgery Pauley Heart Center Virginia Commonwealth University Richmond VA USA; ^2^ School of Pharmacy Virginia Commonwealth University Richmond VA USA; ^3^ Division of Cardiology Pauley Heart Center Virginia Commonwealth University Richmond VA USA

**Keywords:** animal models, cardiovascular biology, cardiovascular disorders, reproducibility

## Abstract

Heart transplantation is a lifesaving procedure, which is limited by the availability of donor hearts. Using hearts from donors after circulatory death, which have sustained global ischemia, requires thorough studies on reliable and reproducible models that developing researchers may not have mastered. By combining the most recent literature and our recommendations based on observations and trials and errors, the methods here detail a sound in vivo heterotopic heart transplantation model for rats in which protective interventions on the ischemic heart can be studied, and thus allowing the scientific community to advance organ preservation research. Knowledge gathered from reproducible animal models allow for successful translation to clinical studies.

## 1 INTRODUCTION

Donation after brain death (DBD) donors are the primary source of donor hearts for transplantation.^1^ Donation after circulatory death (DCD) donors represent an important but underutilized source of additional hearts for transplantation.^1^ The main distinctions between DBD and DCD donors are (a) DCD donors do not have a complete loss of brain function, (b) due to the untreatable and grave nature of illness withdrawal of life support is agreed by the patient or a legal representative to pronounce the person dead, (c) warm ischemia of varying duration based on the cardiorespiratory reserve of donor to all organs including heart is inevitable, and (d) the amount of permanent damage to donor organs and their potential for recovery of function is unknown. Principle reasons for underutilization of DCD hearts are ischemic damage and uncertain viability of heart.^2^

Developing a successful animal model to study the modulation of ischemia and reperfusion (I/R) injury is critical in gathering valuable information to promote DCD heart transplantation. A sound knowledge gathered from animal models would allow for successful designing and testing interventions that would pave the way for clinical studies. Survival rates after heterotopic heart transplantation in rats were extremely variable and poor prior to the seminal publication of murine heterotopic heart transplantation technique in 1969 by Oto and Lindsey.^3^, ^4^ They first described the proper method of heart procurement from donor rats and performed end‐to‐side vascular anastomosis in the recipients. Subsequent to their publication, improvements were made in the choice and delivery of anesthesia (inhalation of isoflurane as anesthetic agent),^5^ limiting dissection of lumbar vessels (to prevent spinal cord ischemia),^6^ and the use of curved vascular clamps to obtain control of infrarenal aorta and vena cava.^6^ These improvements resulted in shorter operative times, decreased complication rates (bleeding, paraplegia), and higher survival rates.^5^, ^6^

We successfully incorporated the improvements mentioned above for murine DBD heterotopic heart transplantation and observed reproducible survival outcomes. However, when we attempted murine DCD heterotopic heart transplantation, we encountered setbacks that challenged us to examine and modify our surgical techniques. For example, unlike DBD hearts, DCD hearts remain stiff, noncompliant, and require more space in the recipient abdomen during vascular anastomosis. In addition, since antemortem cardioplegia cannot be administered per simulated clinical DCD protocol a novel approach was necessary to deliver cardioplegia post‐mortem. Incorporating several modifications as we gathered experience, we were able to perform successful DCD heterotopic heart transplantations. Many of the details we gathered during the learning process were not described in the published literature on murine heterotopic heart transplantation. Our objectives were to describe in detail the challenges encountered in performing DCD heterotopic heart transplantation, possible reasons for failures, and describe the modifications we adopted to overcome the challenges.

Here we describe the characteristics of the DCD process related to donor heart and hemodynamic changes associated with the surgery in the recipient rat. We consider these observations pivotal for successful DCD heterotopic transplantation experimental outcomes. It is our sincere desire that the reader of this manuscript will benefit from our experiences and be able to establish a successful DCD heart transplantation setup with reproducible survival outcomes.

## 2 METHODS

All experimental animals were cared in accordance with institutional guidelines and the Guide for the Care and Use of Laboratory Animals, published by the National Institutes of Health (NIH Publication No. 86‐23, revised 2011).^7^ The following procedures were approved by the Virginia Commonwealth University's Animal Care and Use Program, including the Institutional Animal Care and Use Committee with the Division of Animal Resources, for the ethical treatment of animals under protocol number AD20114. The Sprague‐Dawley rats were housed in a husbandry under controlled humidity, temperature 23°C, and 12 hours dark/light cycles.

### 2.1 Characteristics associated with the DCD donor heart defined in ex vivo experiments

#### 2.1.1 Induction of DCD process

The donation after circulatory death was induced in rats sedated in a 3% isoflurane chamber then anesthetized with ketamine/xylazine (100/10 mg/kg intramuscular). The rats received intravenous injection of heparin (1000 units/kg), were intubated and connected to a ventilator (1 mL/kg at 90 rpm) and received an intravenous injection of vecuronium bromide (4 mg/kg). After one minute, the ventilator was disconnected and the DCD process initiated.

#### 2.1.2 Effects of duration of DCD‐associated ischemia on ex vivo heart function recovery

**FIGURE 1:**
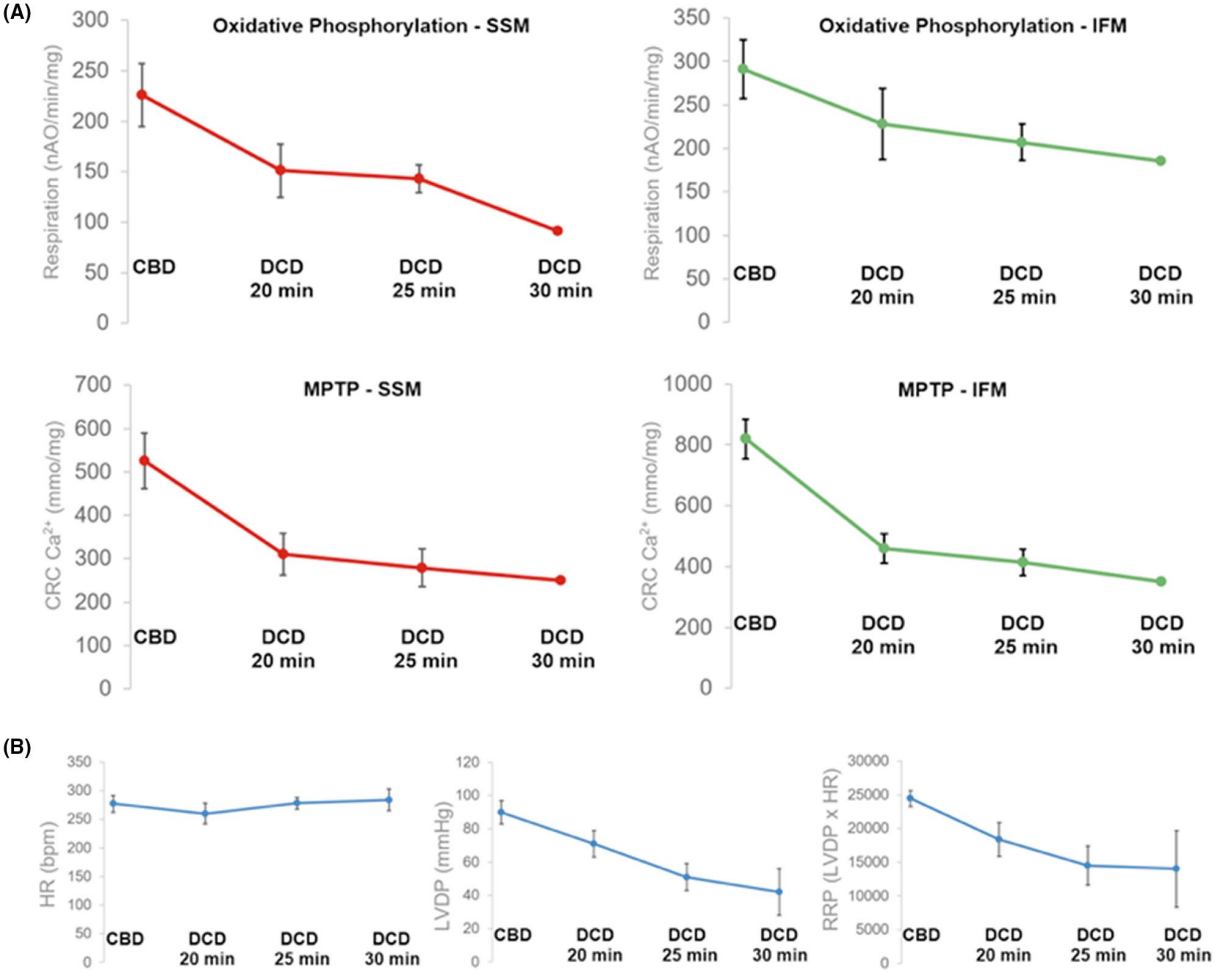
Mitochondrial function (A) and myocardial function (B) of donation after circulatory death (DCD) hearts with 20, 25, and 30 min of warm ischemia compared to control beating donor (CBD) hearts. A significant decline is seen in both oxidative phosphorylation and calcium retention capacity (CRC) in subsarcolemmal (SSM) and interfibrillar mitochondria (IFM). While heart rate (HR) remain comparable between control and DCD hearts with variable periods of ischemia, a steady and significant decline was observed in left ventricle developed pressure (LVDP) and rate pressure product (RPP = HR × LVDP). MPTP = mitochondrial permeability transition pore, Sample size CBD n = 8, DCD 20 min n = 5, DCD 25 min n = 8, DCD 30 min n = 4

Duration of ischemia is a critical component of the DCD damage, and myocardial injury is proportionate to the duration of ischemia. To determine the maximum duration of ischemia for DCD murine hearts with reversible damage, we examined the loss of myocardial function in rat hearts with 20, 25, and 30 minutes of warm ischemia by reanimating the hearts on a Langendorff system in preparation for standardizing procedures for heterotopic transplantation (Figure 1). We noticed that with a shorter DCD ischemic duration (15 minutes), the rat hearts did not show a significant decrease in myocardial contractile function or oxidative phosphorylation in isolated mitochondria. The loss of myocardial function at 25 minutes of ischemia was between 40% and 45% of the control rat hearts, which improved with time on the Langendorff perfusion. With an ischemia time of 30 minutes, the loss of myocardial function was over 50%, and further deteriorated on the Langendorff perfusion system with time. We, therefore, identified 25 minutes of ischemia in rats as the maximum length of time that results in significant but reversible injury to the hearts to model DCD transplantation conditions.

To further determine maximum warm ischemic time, cardiac activity was followed using echocardiography and continuous electrocardiography, with two electrodes placed in the upper limbs and one in the hind limb after general anesthesia. A cannula was placed within the carotid artery for continuous blood pressure monitoring and serial blood gas values. Within 2 minutes of withdrawal from the ventilator, systolic blood pressure would fall below 50 mmHg with a blood oxygen saturation less than 70%. Within 10 minutes of withdrawal from the ventilator, electric asystole was observed which marks the beginning of the 5‐minute standoff period required after asystole prior to initiating procurement.

For heterotopic heart transplantation, it takes approximately 5‐7 minutes to procure the heart from the donor rat and administer cold cardioplegia. To achieve the target of 25 minutes of ischemia, we propose to start the procurement of the rat heart 18 minutes following the termination of ventilation, to allow a maximum time of 25 minutes of warm ischemia which includes standoff time. We suggest that the start of procurement can be changed based on the experience of the person performing the procurement.

#### 2.1.3 Effects of donor age and weight on ex vivo reanimation

Young rats tolerate ischemia to myocardium better than older rat hearts. It is our observation that rats older than 16 weeks are likely to sustain irreversible damage with 25 minutes of DCD ischemia. To maintain a homogeneity based on our limited observations with different ages and weights of rats, we suggest conducting DCD studies in rats of ages between 8 and 16 weeks weighing less than 400 g.

#### 2.1.4 Effect of gender

Differences based on gender were not studied in our experiments. We only used male rats for our studies, and our results need to be interpreted with this limitation. We recommend defining the maximum tolerability and the appropriate age/weight groups to use in experiments designed to use female rats as DCD heart donors.

### 2.2 Effects of the heterotopic heart transplantation surgery on the recipient

#### 2.2.1 Measurement of hemodynamic parameters

As a first step to better understand the reasons for lack of recipient survival after DCD heart transplantation, we chose to monitor the hemodynamic changes occurring in the recipient rats (n = 4) at the time of surgery and immediately following reperfusion of the transplanted heart. To accomplish this, we cannulated the carotid artery with a polyethylene tube (0.8 mm external diameter) filled with normal saline (0.9% NaCl) and connected to a pressure probe and a power lab station (AD Instruments, Denver, CO) to transduce continuous blood pressure recording. We used the same carotid access to collect timed blood samples corresponding to the steps of heterotopic heart transplantation and define the impact of bleeding, lactic acidosis, hyperkalemia, hypocalcemia, and hypoxia. These parameters were measured using 0.2 mL of blood with the ABL‐800 blood gas analyzer (Radiometer, Copenhagen, Denmark). Core temperature was carefully maintained between 37°C and 38°C using a water‐heated pump with a therapy pad (Adroit Medical Systems, Loudon, TN). The following observations were made from the above‐described monitoring protocol.

1. Systolic blood pressure increased by 20 mmHg from baseline after clamping the abdominal aorta and vena cava. With ongoing surgery, it returned to baseline in 5‐7 minutes.
2. Systolic blood pressure decreased by 20‐30 mmHg upon releasing the aortic and vena cava clamp following completion of graft anastomosis. In the absence of bleeding from anastomosis sites, blood pressure improved in less than 5 minutes and attained baseline status in ten minutes.
3. Hematocrit at the start of transplantation was between 33% and 36%. It remained steady during the surgery, but after releasing the clamp on the aorta and vena cava, the hematocrit dropped by 7‐10 points. If hemostasis was not attained in the first five minutes, the hematocrit continued to drop below 20%. Which invariably led to the demise of the recipient rat.
4. Electrolytes, and in particular potassium levels, remained steady during the procedure and immediately after reperfusion of transplanted heart. Hyperkalemia was not detected in any of the four rats.
5. Lactic acid levels remained normal and steady during the procedure and immediately after the reperfusion of transplanted heart.
6. Oxygen content in the arterial blood samples remained over 150 mmHg with 3% isoflurane inhalation anesthesia for induction and 2% for maintenance. The gas anesthesia mix was 1.5%‐3% isoflurane and the rest (98.5%‐97%) oxygen.
7. Carbon dioxide levels continue to increase from 40 mmHg at the baseline to over 60 mmHg by the end of surgery. There was a corresponding respiratory acidosis but no metabolic acidosis.

#### 2.2.2 Examination for vascular anastomosis patency and thromboembolism

Twenty‐four hours following transplantation, we euthanized the rats and then performed dissection of the aorta and inferior vena cava to look for patency of vascular anastomosis and recipient pulmonary artery to look for evidence of thromboembolism; the vascular anastomoses were patent and no clots were found in the recipient pulmonary artery. Following the anti‐coagulation protocol, we recommend that we describe to prevent any thrombus formation in the anastomosis.

From the observations listed above and modifying the techniques that were described in the published literature, we describe here the factors that lead to a successful heterotopic DCD heart transplantation (Tables 1 and 2).

**TABLE 1:** Summary of recommendations for murine heterotopic donation after circulatory death heart transplantation

Category	Recommendation
Species	Sprague‐Dawley (other species not studied)
Age	8‐16 wk
Weight	<400 g
Gender	Male (females not studied)
Temperature	38°C
Maximum warm ischemia duration	25 min
Cardioplegia access	0.8‐mm tube with 3‐way stop cock in the right common carotid artery
Aortic division	Immediately proximal to the innominate take off.
Pulmonary artery division	At the bifurcation
Pulmonary vein and vena cavae division	Ligate securely with 4‐0 silk, hand ties prior to division
Anesthesia of recipient	Inhaled isoflurane: 3% for induction, 2%‐2.5% for maintenance, 1.5%‐2% for the final 5 min before the closure of abdomen. Long‐acting analgesia for postoperative care, administer 5 min before completing the surgery.
Anticoagulation	Donor: 1000 U/kg through tail vein, 100 U through carotid access Recipient: None. Heparinized saline flush when entering vessels
Dissection of abdominal vessels in recipient	Only anterior exposure using a cotton‐tip swab, partial occlusion vascular clamp for limited isolation for anastomoses
Suture for anastomoses	8‐0 monofilament on a 4‐mm taped needle, 5‐7 cm length of working suture, starting at 1 o'clock
Hemostasis	Surgicel^®^
Fluid Management	3 mL prior to pulmonary artery anastomosis, provide more as needed for insensible loss

**TABLE 2:** Solutions list for murine heterotopic donation after circulatory death heart transplantation

Consumable solutions
Isoflurane, USP (Patterson Veterinary, NDC 14043070406)
Vecuronium Bromide diluted in PBS for 100 mg/mL (Sigma‐Aldrich, cat. No. PHR1627)
Ketamine HCl 100 mg/mL (Henry Schein, NDC 6745710810)
Xylazine 100 mg/mL (Pivetal Anased, NDC 04606675002)
Belzer UW^®^ cold storage solution Pentafraction 50 g/L Lactobionic acid (as Lactone) 35.83 g/L Potassium phosphate monobasic 3.4 g/L Magnesium sulfate heptahydrate 1.23 g/L Raffinose pentahydrate 17.83 g/L Adenosine 1.34 g/L Allopurinol 0.136 g/L Total glutathione 0.922 g/L Potassium hydroxide 5.61 g/L Sodium hydroxide/hydrochloric acid to adjust the solution to pH 7.4
Normal saline
Lidocaine HCl 2% (Aspen Vet, Patterson Veterinary No. 07‐892‐4325)

### 2.3 Optimization of the DCD heart procurement and preparation

#### 2.3.1 Donor preparation

An isoflurane chamber was used for sedating the rats, and then ketamine/xylazine (100/10 mg/kg) was administered intramuscularly prior to shaving the fur of the neck and chest. The skin was cleansed with sterile alcohol wipes and povidone solution. Heparin (1000 U/kg) was administered intravenously via tail vein with a 25G needle.

#### 2.3.2 Cardioplegia requirement and delivery in a DBD heart vs DCD heart

The literature on rat heart transplantation routinely mentions the need for cardioplegia.^5^, ^6^, ^8^ Delivery of cardioplegia to a DBD heart protects the myocardium from ischemia and prevents the heart from stiffening during storage. It also washes off the remaining blood from the coronaries. Cardioplegia is most commonly administered through cannulation of the inferior vena cava (IVC) while the heart is still beating and occasionally delivered directly into the aorta with a 25G needle before the procurement of the heart. These delivery methods are non‐physiologic or carry a serious risk of damage to the ascending aorta. Since there is no ongoing circulation in DCD donors, the above‐described cardioplegia delivery methods are not practical. Direct access to the ascending aorta is possible in DCD hearts, but a significant risk of damage to the aortic valve or ascending aorta, which negatively affects the heart transplantation procedure, precludes its practice. In our laboratory, we developed a method of delivering cardioplegia to a donor's heart via right carotid artery cannulation, using a polyethylene tube with an external diameter of 0.8 mm, which has been effective in our practice. The following is a detailed description of our approach.

#### 2.3.3 Donor preparation and induction of the DCD process

**FIGURE 2:**
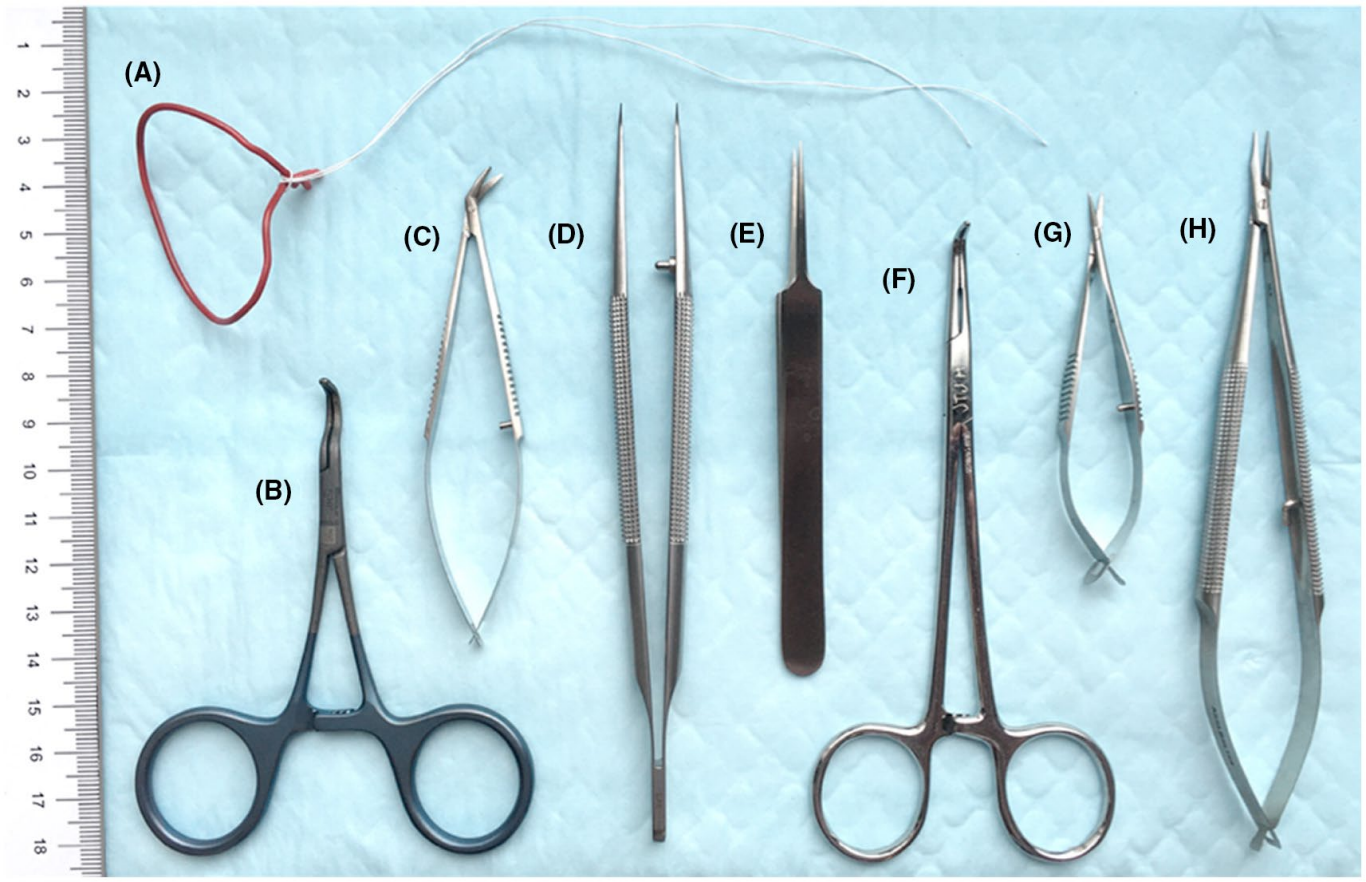
Microvascular instruments used for heterotopic rat heart transplantation. From left to right: A, Curved hand‐molded abdominal wall retractors, using large coated paperclips on suture. B, ebakey atraumatic pediatric multi‐angle vascular clamp (Aesculap^®^ cat. No. F341T). C, icro‐scissors, right angle and curved tips (Braintree Scientific Inc, cat. No. SC‐MS 154). D, Forceps with curved tips (ASSI^®^ cat. No. ASSI.228). E, Tweezers with high precision point (Excelta™ 5SA, cat. No. 17‐456‐109). F, Right angle hemostat (ASSI^®^ cat. No. ASSI.AG49626). G, Iris micro‐scissors with straight tips (ASSI^®^ cat. No. ASSI.5253). H, Needle holder with the lock mechanism removed (ASSI^®^ cat. No. ASSI.BSL158)

Aseptic approach was practiced with donor and recipient animals routinely. Key instruments used are further described in Figure 2.

**FIGURE 3:**
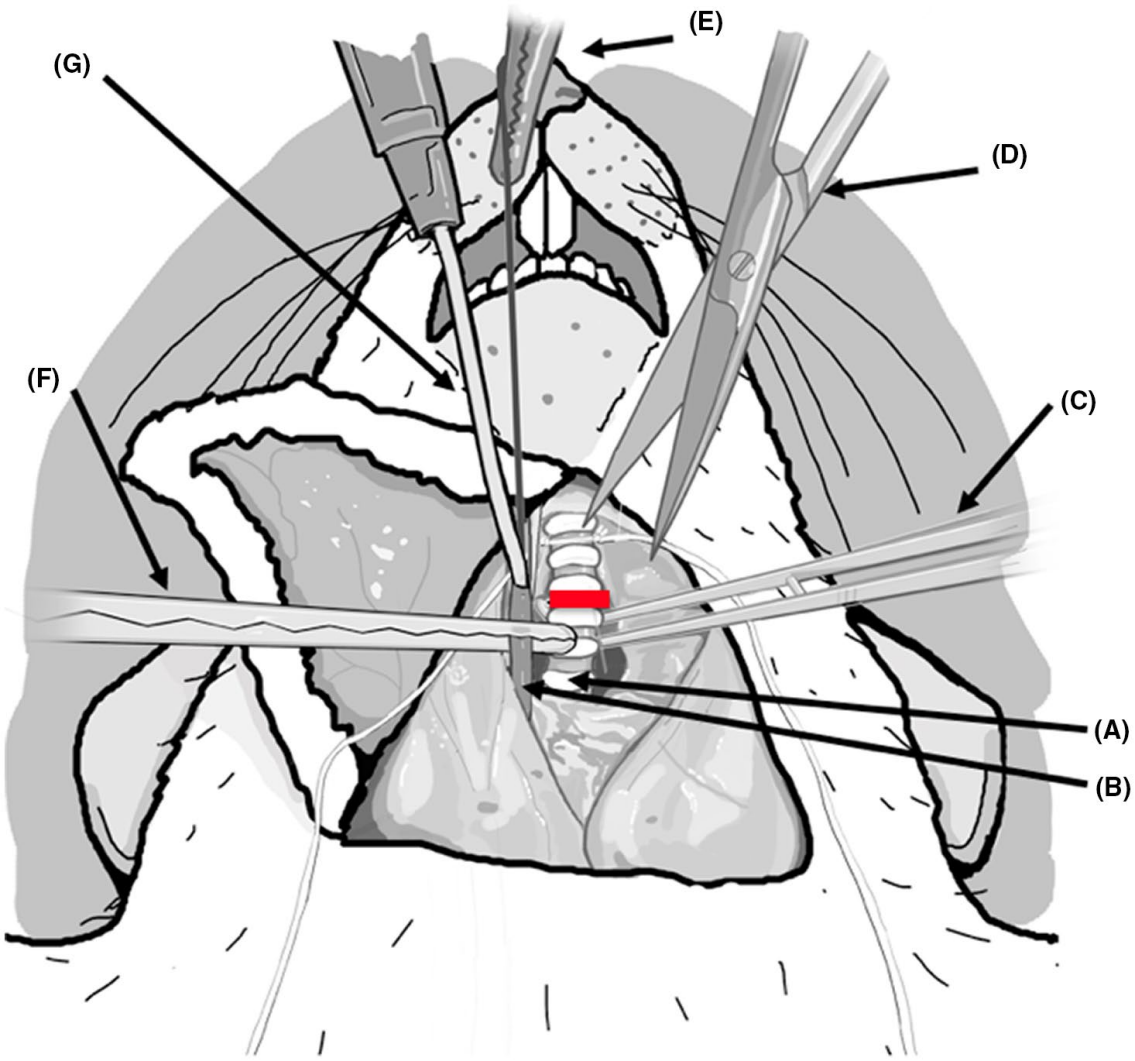
Cannulation of the right common carotid artery and endotracheal intubation: Under general anesthesia, the anterior neck was exposed, strap muscles of the neck were retracted to expose trachea (A) in the midline and common carotid artery (B) to its left (as seen in the picture). The trachea is exposed on a curved iris forceps (C) and opened anteriorly with fine scissors (D) between the tracheal rings (red line). A 14‐gage angiocath attached to the ventilator is inserted into the trachea and secured with a 4‐0 silk tie. A 4‐0 silk tie on the cranial end of carotid artery for traction (E) and proximal control with atraumatic vascular clamp (F) allows for insertion of a 22‐gauge soft tube (G) into the carotid artery which is secured with a 4‐0 silk tie

1. Place the rat in a supine position on a heating pad with the head near the operator. Make a wide inverse V‐shaped incision with scissors on the neck, with “V” pointed toward rat's chin. Once the skin is cut and flipped toward the chest, bluntly separate the midline neck strap muscles (sternomastoid and sternohyoid) with forceps to identify the glottis and tracheal rings (Figure 3).
2. Place a 4‐0 silk tie around the trachea in preparation for cannulation and keep it aside. If the trachea is cannulated early at this point, it becomes stiff from the endotracheal tube and will not retract easily during carotid dissection (Figure 3).
3. Immediately lateral to the glottis lies on the right side is the common carotid artery and vagus nerve, as they run together parallel to the trachea. Carefully isolate the common carotid artery to the full length of the neck.
4. Tie off the distal (cranial) end of the carotid artery with 4‐0 silk and attach a hemostat to the free end of the tie for traction; this will facilitate cannulation of the carotid artery. After mobilizing the proximal‐most aspect of the carotid artery (toward the base of the neck), clamp it with a vascular hemostat (Figure 3).
5. Open the carotid artery with micro‐iris scissors under an operating microscope by partially dividing it anteriorly in the distal‐most end. Cannulate the carotid artery with a beveled end 0.8 mm polyethylene tube into the artery and secure it with a 4‐0 silk tie (Figure 3). A three‐way flow stopcock adopter at the proximal end of the tube allows for easy blood draws and delivery of drugs or cardioplegia solution into the carotid artery as needed. When the proximal vascular clamp on the carotid artery is opened, a pulsatile backflow of blood into the catheter should be seen.
6. Once the catheter is secure in the carotid artery, deliver an additional dose of heparin (100 units) to keep the catheter from clotting.
7. Now expose the tracheal rings again by retracting the strap muscles and stabilizing the trachea with curved fine‐tip forceps. Open the trachea with micro‐iris scissors by partially dividing the trachea between the cartilaginous rings. Cannulate with an endotracheal tube and secure with the previously placed 4‐0 silk. Connect the endotracheal tube (a 14G angiocatheter) to the ventilator and start ventilation (1 mL/kg tidal volume, 90 bpm).
8. To induce the DCD process, a necessary step is to deliver vecuronium bromide (4 mg/kg), a paralyzing agent, via the carotid artery and allow it to circulate for 2 minutes. Monitor the appropriateness of the anesthesia depth to determine if the animal is distressed while paralyzed. We monitor the blood pressure through the carotid cannula. If signs of animal distress are observed, additional anesthesia needs to be delivered.
9. The next step in a DCD heart procurement is withdrawal of the ventilator. Observe for the absence of respiratory activity. The DCD ischemic time starts from the time the ventilator support is withdrawn. If the DCD time is set for 25 minutes of maximum ischemia, then start dissection at 20 minutes from the time the ventilator was discontinued to allow at least 5 minutes to perform the heart procurement. We recommend setting the time based on the experience of the operator and we began dissection after 18 minutes to ensure completion within 25 minutes.

#### 2.3.4 Heart procurement and cardioplegia administration via the right common carotid artery

**FIGURE 4:**
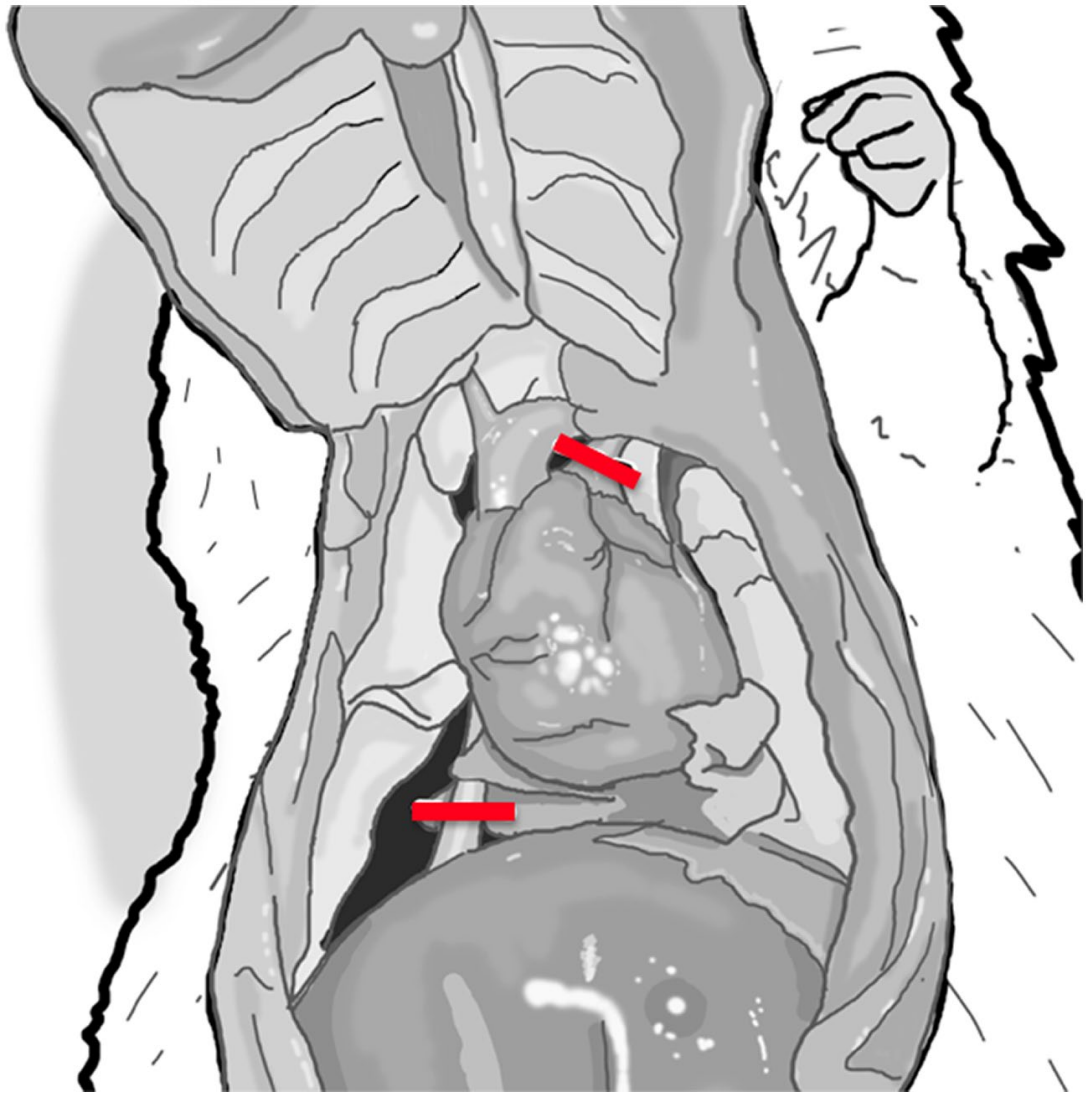
Bilateral anterolateral thoracotomy exposes the heart and great vessels. To decompress the heart, pulmonary artery (red line in upper part of picture) and inferior vena cava (red line in lower part of picture) can be transected

**FIGURE 5:**
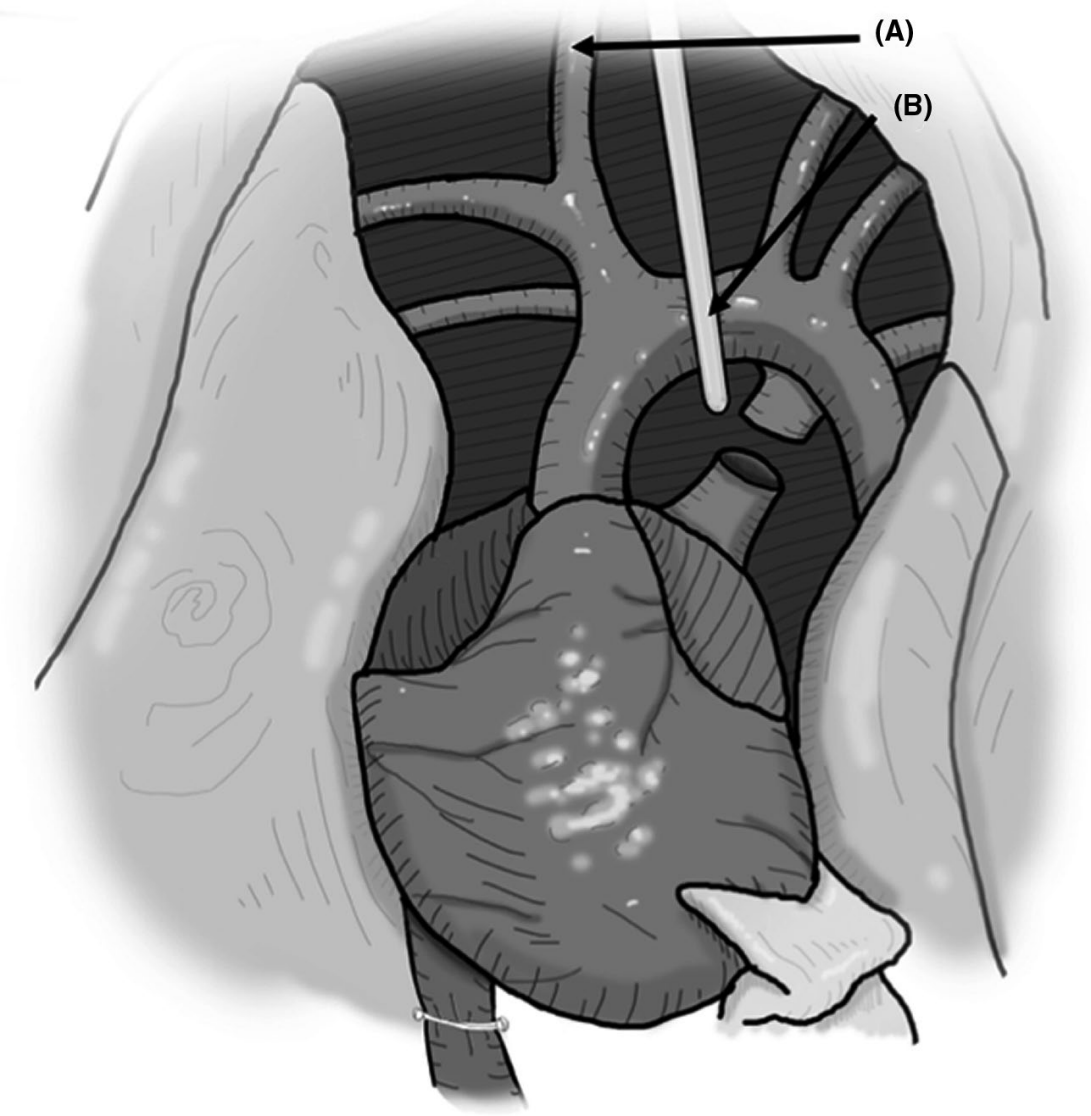
Focused view of aortic arch. Prior to injecting cardioplegia into the right common carotid artery (A), aorta (B) is clamped distal to the takeoff of innominate artery allowing the flow of plegia solution into the coronaries

1. Divide the abdominal wall at the costal margin starting from the level of xyphoid, and then perform bilateral anterior thoracotomies by dividing the ribcage parallel to the sternum on either side up to the clavicles. Be careful to keep the thoracotomy (ribs) division close to the sternum (Figure 4). If too lateral, the divided edges of ribs can puncture and damage the heart surface during the dissection. The cut edges of ribs optionally may be covered with surgical gauze to protect the heart from being punctured by sharp edges of the divided ribs. Care should be taken not to damage the internal thoracic arteries or arteries in the thymus tissue since damage to these vessels will lead to leakage of the cardioplegia that is planned to be delivered later. This part of the dissection is best performed with an operating microscope under low magnification (X 5) that provides good visualization and a larger field of vision.
2. To decompress the heart chambers, encircle the inferior vena cava (IVC) with a 4‐0 silk and then divide the IVC using micro scissors close to the dome of the liver (Figure 4). Blot away the venous blood with surgical gauze. With the heart decompressed, it is easy to dissect the plane between the ascending aorta and the pulmonary artery (PA) with blunt dissection. A small amount of fat and connective tissue lies between the aorta and pulmonary trunk. This needs to be cleaned with micro‐iris scissors and blunt tip forceps, taking care not to damage either of the main blood vessels or remove too much of the adventitia from the ascending aorta.
3. Isolate the pulmonary trunk with blunt dissection using the curved fine‐tip forceps to its bifurcation into right and left main pulmonary arteries. Carefully divide the pulmonary artery close to its bifurcation point, avoiding any damage to the left atrial appendage, which overlaps part of the proximal pulmonary trunk (Figure 4).
4. Clear away the tissue under the aortic arch with blunt dissection to allow access for a small right‐angle vascular clamp to be placed across the aortic arch distal to the origin of the innominate artery (Figure 5).
5. Once the IVC and PA are divided, and the heart is decompressed. Clamp the arch between the innominate artery and the common carotid (Figure 5) and manually deliver cardioplegia (10 cc of University of Wisconsin solution at 4°C over 2‐3 minutes) through the carotid access, while watching the aorta under a microscope for over‐distention.
6. The timing of cardioplegia delivery in a DCD heart should correspond to the preset ischemia duration. For example, 25 minutes since the termination of ventilation for a study designed with 25 minutes of ischemia.
7. Following cardioplegia delivery, tie off the IVC toward the heart with 4‐0 silk (Figure 5). Next, divide the aorta with 45° angle micro‐scissors.
8. Lastly, ligate the pulmonary veins and both superior vena cavae (SVCs) together with 4‐0 silk. Since these ties hold a large amount of tissue, we recommend securing this firmly, optimally with a hand tie instead of an instrument tie. Bleeding from a loose pulmonary vein is very difficult to control following transplantation. The atrial appendage tissue tends to bunch into this suture. Using a cotton‐tip swab to gently pull the heart down toward abdomen while tying the pulmonary veins. This will keep the atrial appendages away from the tie. Once the tie is secured, divide the pulmonary veins with micro‐iris scissors and collect the heart from the donor rat.
9. Immediately place the heart in the cold saline solution at 4°C. A well‐protected heart will be soft at touch, pale from having all the blood flushed from the coronaries, and cold. The DBD hearts are soft, while DCD hearts tend to be firmer.

#### 2.3.5 Preparation of recipient and heterotopic heart transplantation

**FIGURE 6:**
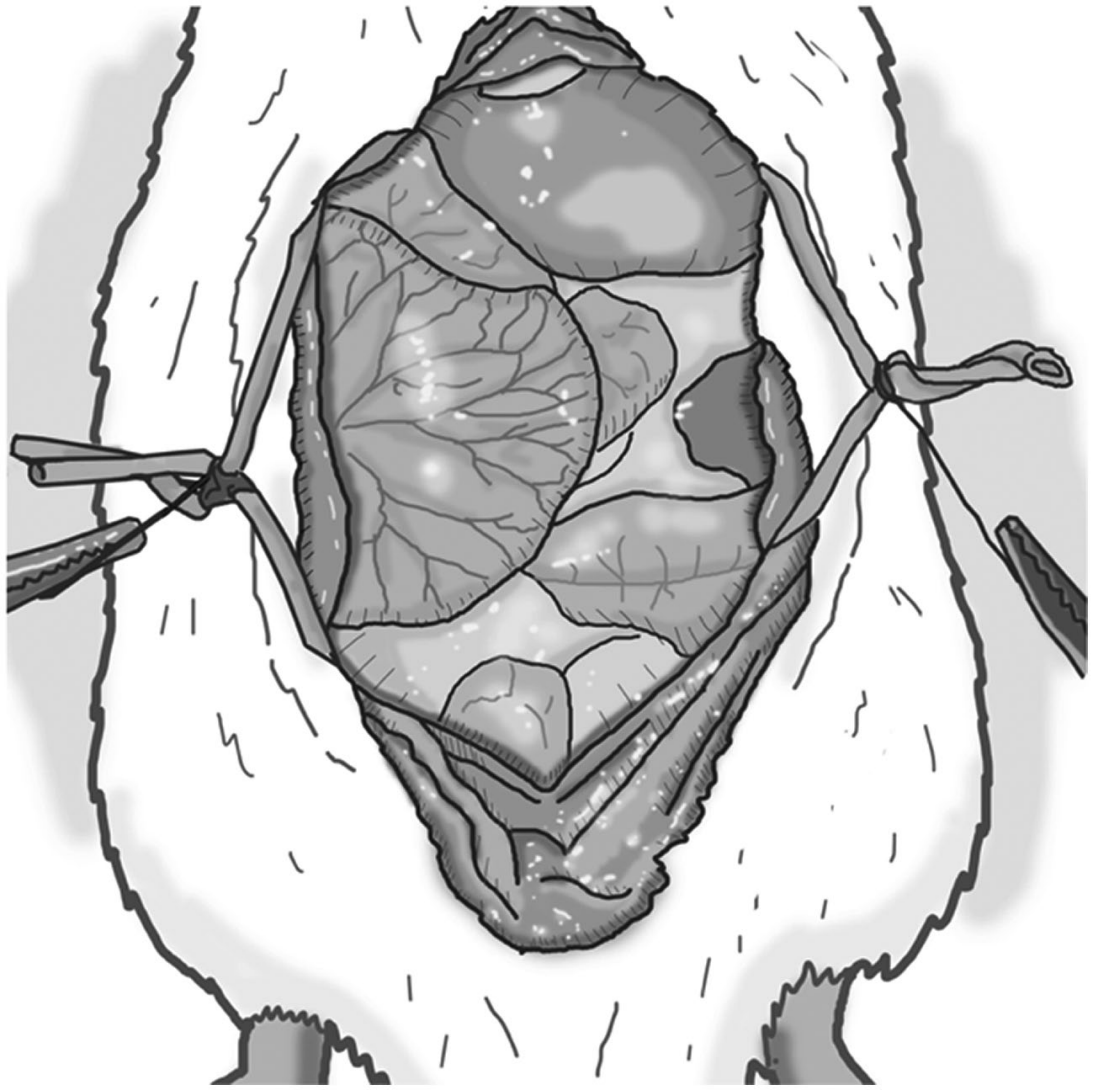
Midline laparotomy and exposure of viscera with curved hand‐molded abdominal wall retractors

**FIGURE 7:**
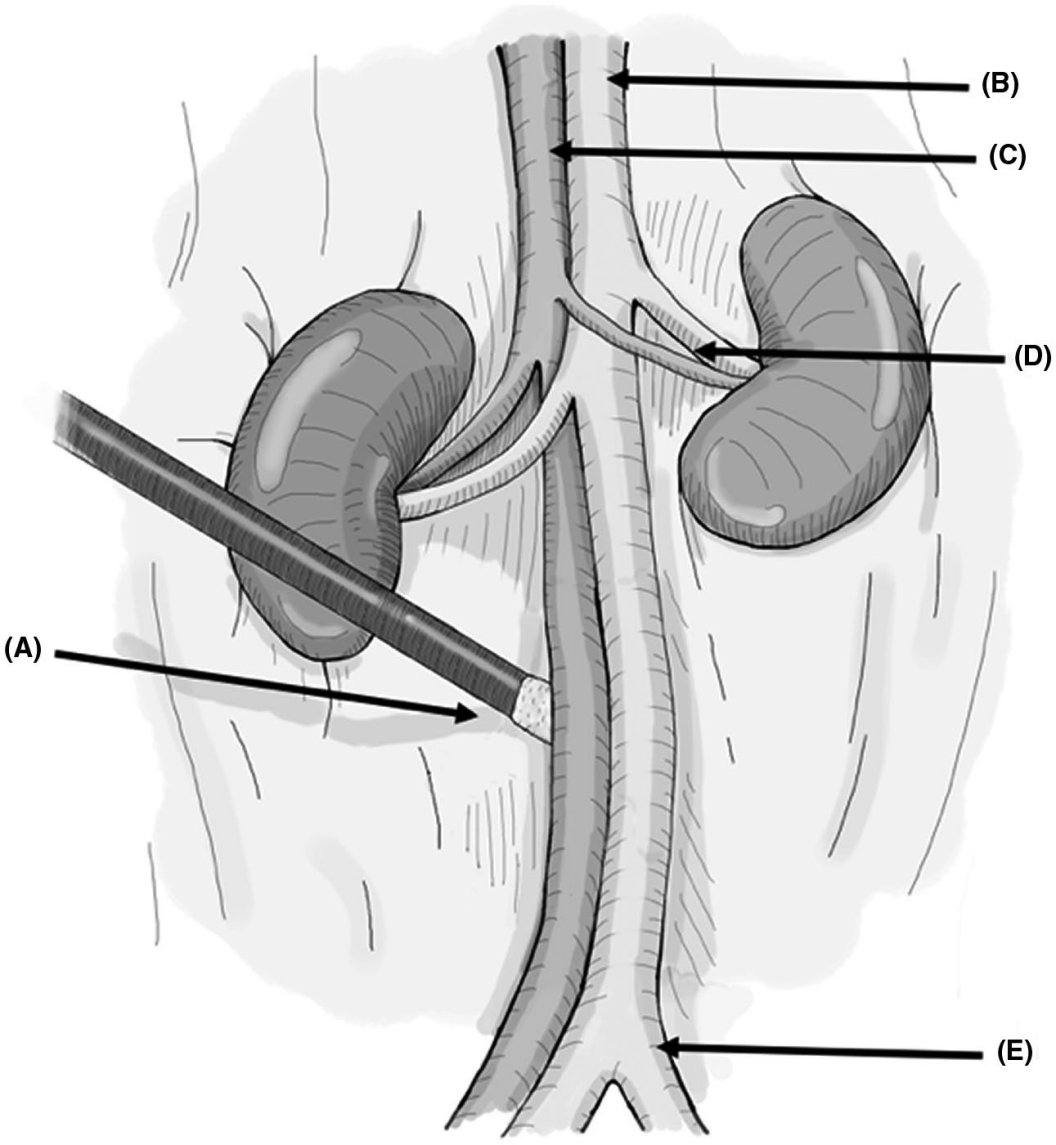
Recipient abdominal aorta and inferior vena cava exposure for heterotopic heart transplantation. Following midline laparotomy and displacement of bowel, retroperitoneum is exposed. Using cotton tip swabs (A) gently open the retroperitoneum overlying the aorta (B) and inferior vena cava (C). Sweeping aside the retroperitoneal fat, clear aorta and inferior vena cava from renal pedicles (D) to aortic bifurcation (E)

**FIGURE 8:**
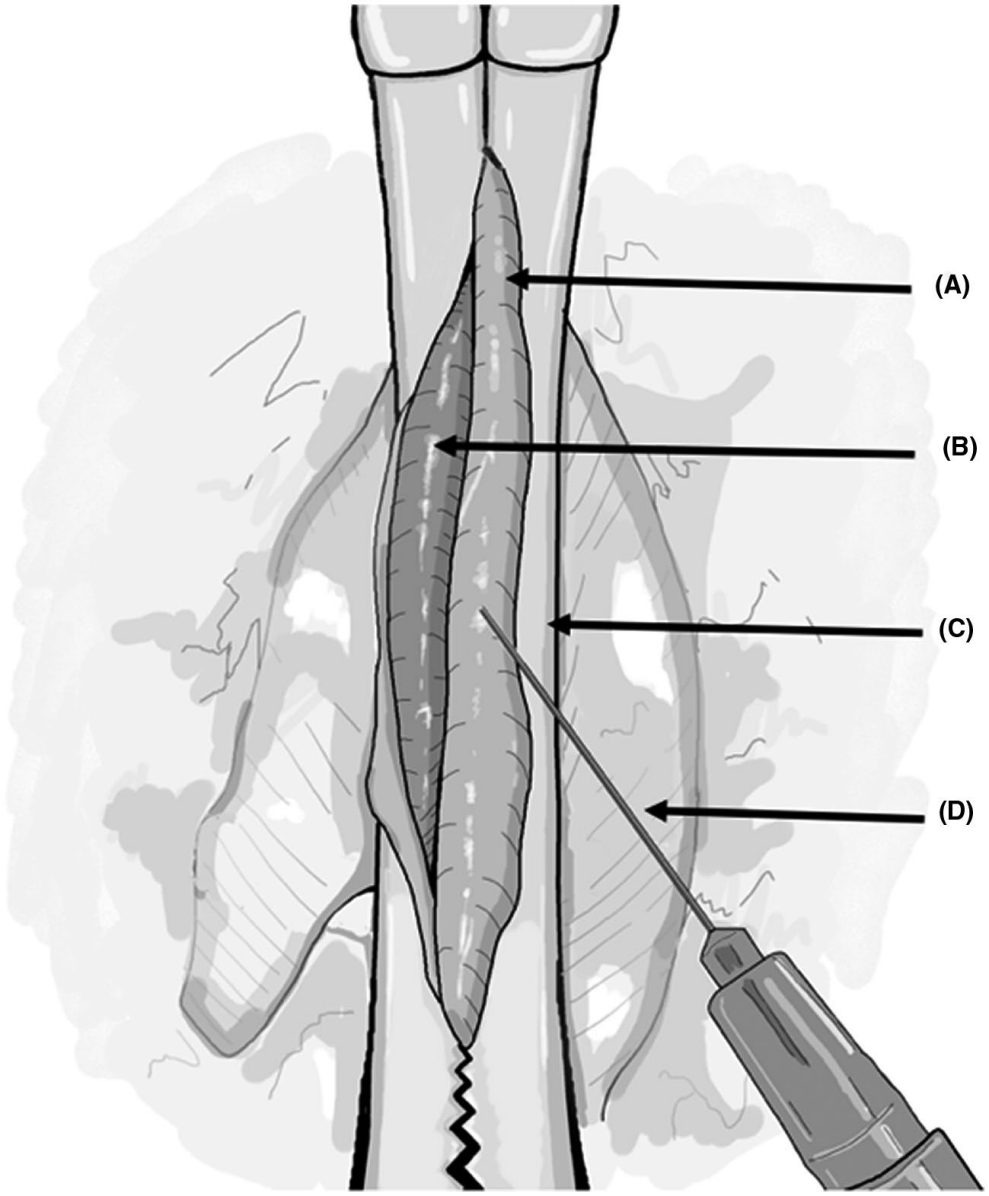
Isolating the aorta (A) and inferior vena cava (B) with the atraumatic vascular clamp (C). Once the vessels are isolated, a 30 gage (D) needle is used to open the vessels, first by injecting the heparinized saline then extending the arteriotomy/venotomy

**FIGURE 9:**
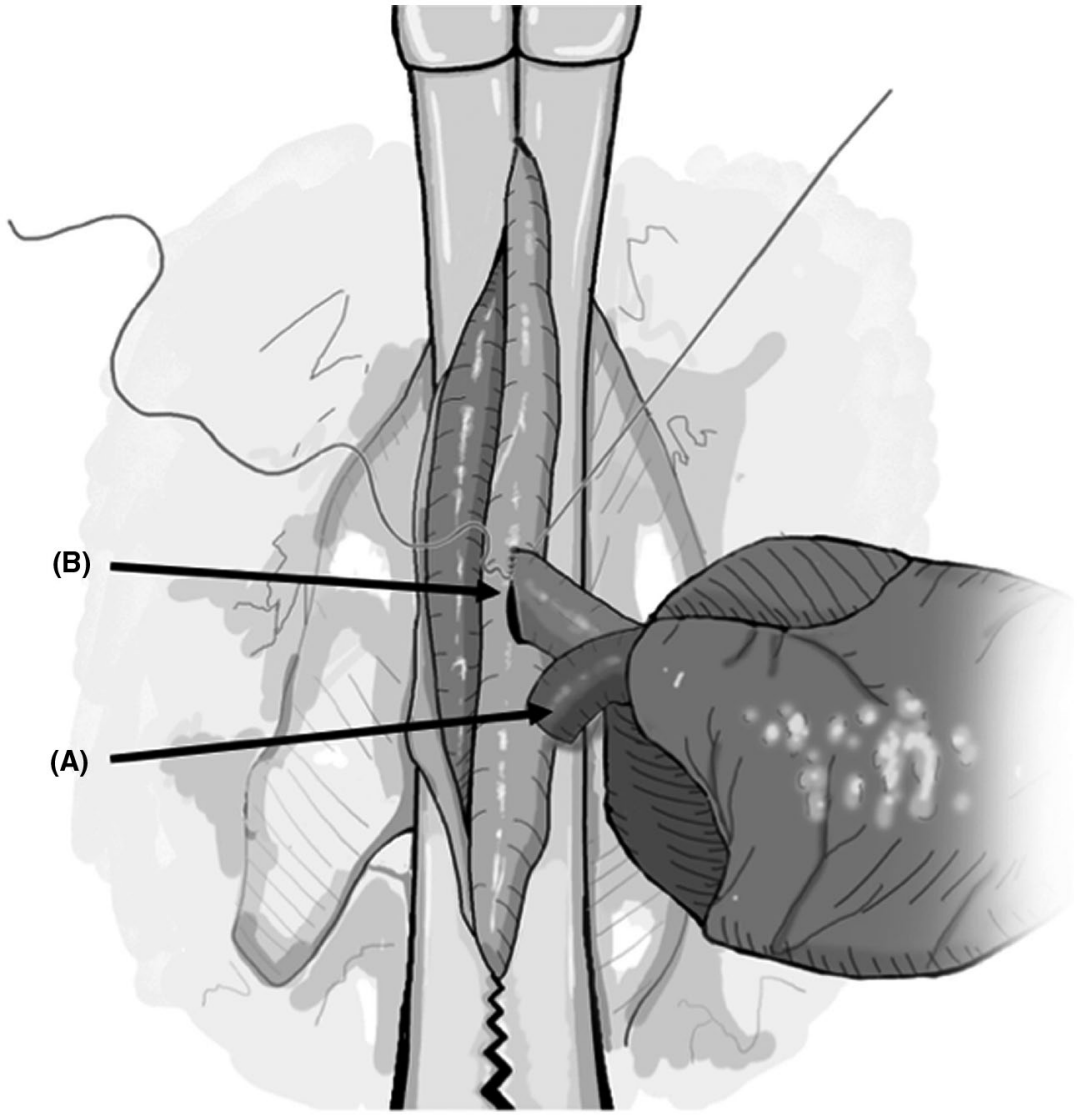
Aorta and pulmonary artery anastomosis. Donor heart is oriented to the right of the recipient abdominal vessels with donor pulmonary artery (A) lying anteriorly. An end to side anastomosis is performed with donor aorta to the recipient abdominal aorta (B), followed by end to side anastomosis between donor pulmonary artery and recipient inferior vena cava

**FIGURE 10:**
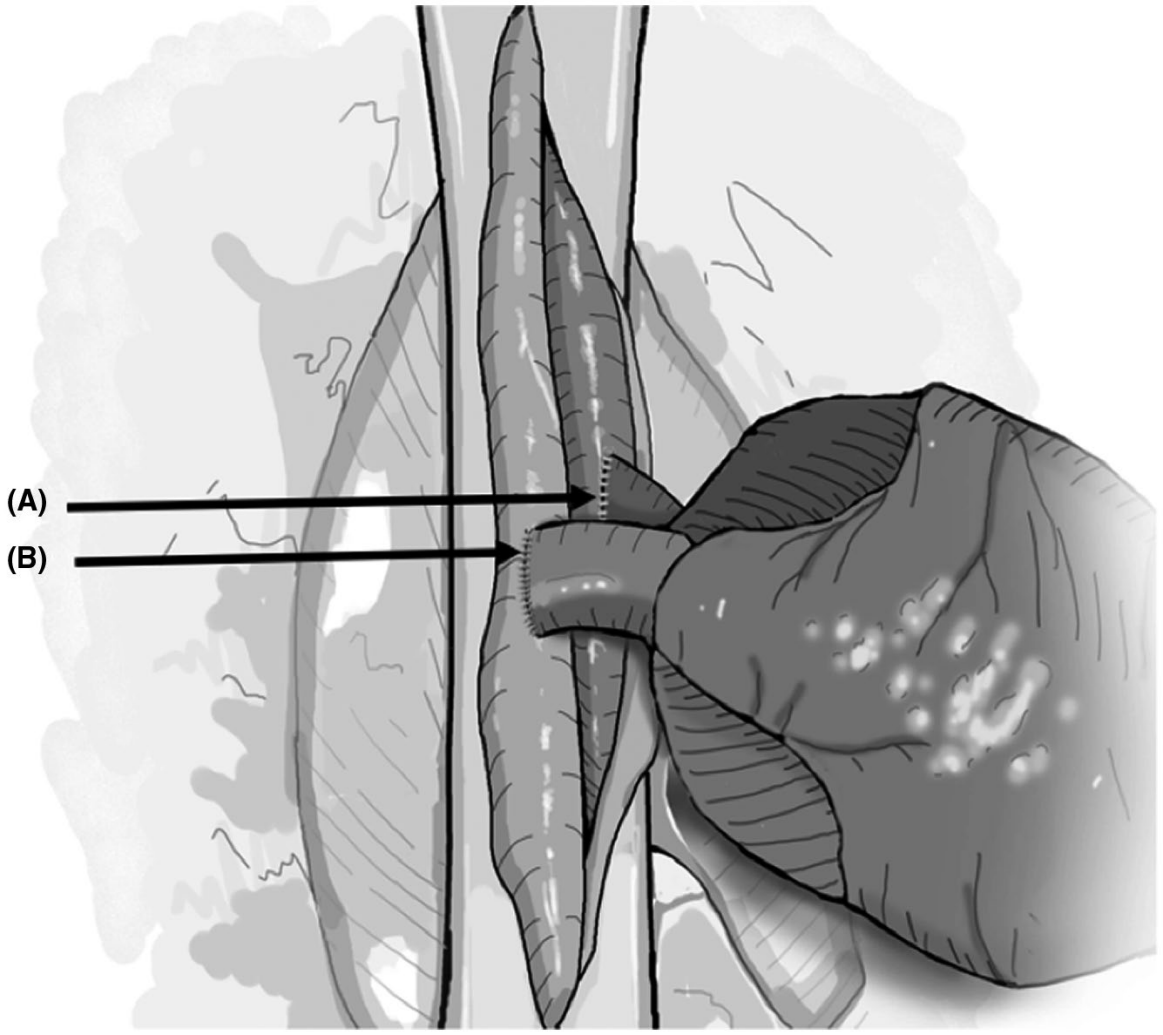
Completed anastomosis. Donor aorta to recipient aorta (A) and donor pulmonary artery to the recipient inferior vena cava (B)

1. Sedate the recipient in an isoflurane chamber (3% induction), then clip hair and cleanse the abdomen with a povidone solution and sterile alcohol wipes. Do not anticoagulate the recipient. If anticoagulated, the suture line of the anastomosis will not stop bleeding from the needle holes.
2. Place the rat supine on a heating pad and monitor the temperature with a probe placed between the rat and heating pad or with a rectal probe. It is critical to maintain the rat's body temperature at 38°C. Two 50‐mL pre‐sterilized centrifuge tubes containing warm saline or glass beads are placed on either side of the abdomen to keep the rat warm. Despite these measures, if the body temperature decreases below 37.5°C, cover all exposed areas of the body with sterile gauze/towels, or autoclaved aluminum foil, leaving just enough area to perform abdominal surgery. Increase the room temperature to prevent further heat loss. Do not tape/secure the limbs of the rat to the surgical field to avoid traction‐related injuries.
3. General anesthesia is induced with 3% isoflurane via a nose cone and then decreased to 2.0%‐2.5%.
4. Open the abdominal skin with scissors in the midline from the xyphoid to the lower abdomen, and then open the abdomen following the linea‐alba, as it is the least vascular plane of the abdomen.
5. Following laparotomy, place abdominal wall retractors to expose working space (Figure 6). A curved hand‐molded broad‐based retractor using large coated paperclips attached to 4‐0 silk suture on hemostats provides good exposure. Resting the hemostats over the two 50 mL centrifuge tubes on either side of the abdomen also allows the retractors to lift the abdominal wall up and out, creating more room for the donor's heart to be placed inside the abdomen.
6. Eviscerate dilated or full colon and place it in a warm moist gauze to the left of the operator, periodically irrigating with warm (38°C) saline. This provides more space, especially for the DCD hearts, as they are very stiff and require more room to accommodate in the abdomen. In the DBD hearts, this additional space may not be required. Of note, leaving bowel outside the abdomen for prolonged durations may cause significant metabolic acidosis from splanchnic ischemia. If bowels are kept out, then keep close track of time and periodically irrigate with warm (38°C) saline on the gauze covering the bowel.
7. To open the retroperitoneum in the midline, use cotton‐tip swabs to expose the aorta and IVC from below the takeoff of the renal vessels to above the aortic bifurcation (Figure 7). Gentle dissection is critical, as the renal artery could be very small, especially an aberrant or accessory renal artery.
8. Isolate 2.5‐3 mm of the infra‐renal aorta and IVC for anastomosis. It is not necessary to dissect them circumferentially as the Cooley vascular clamp will isolate the aorta and IVC for anastomosis. Avoiding circumferential dissection behind the IVC and aorta preserves lumbar vessels, which are at risk of injury leading to bleeding or paraplegia. Since the IVC is slightly behind the aorta, it needs to be gently lifted up with pickups prior to applying the Cooley vascular clamp. Vessels are prone to vasospasm with blunt dissection; to relax the vessels and thereby improve exposure, two drops of 2% lidocaine may be placed on the vessel after completing the dissection and prior to clamping (Figure 8). In our experience, from the induction of anesthesia to occlusion of the aorta and IVC, will take 5‐7 minutes.
9. Entering the aorta with a 30G needle mounted on a 1cc syringe filled with 100 units of heparin/mL of saline is the safest way to open and flush the vessel. Use pointed tipped forceps to grasp the needle tip under a microscope at the entry point for precision, prevent it from damaging the back wall (Figure 8). After entering the vessel, flush with 0.2‐0.3 mL of the solution. Dab the excess solution with cotton‐tipped swabs as heparin may be absorbed and might cause bleeding from suture lines. The IVC is not opened at this point, and no attempt should be made to create a plane between IVC and aorta, as this will lead to formidable bleeding.
10. Proper orientation of the donor heart during anastomosis is critical. The donor heart can be positioned horizontally, perpendicular to the axis of the recipient aorta and IVC, or at an oblique angle. In both situations, the anterior surface of the donor heart is facing upward so the operator is visualizing the anterior surface, and the donor aorta is slightly lower than the pulmonary artery (Figure 9). We prefer the oblique orientation. With a horizontal orientation, we notice excess tension on the pulmonary artery anastomosis. With oblique orientation, the apex of the donor heart will be pointing to the right of the operator, and pulmonary artery anastomosis is relatively higher to the aortic anastomosis, resulting in less tension on the pulmonary artery anastomosis.
11. Prepare to perform anastomoses with 8‐0 monofilament suture on a tapered 4mm needle loaded on a Castroviejo needle holder with fine tips and the lock removed to prevent accidental trauma to tissues when locking and unlocking. The pointed tip needle driver and precision tweezers with high precision tips are preferred instruments for microvascular anastomosis.
12. We recommend first placing a stay suture at 6 o'clock position on aortic anastomosis to provide for a symmetrical and hemostatic suture line. Once a stay suture is placed then we start the anastomosis at 12 o'clock position with outside‐to‐inside on the donor aorta and inside‐to‐outside on the recipient aorta (Figure 9). Since this suture carries the most tension, a double square knot, also referred to as a surgeon's knot, is preferred. Travel counter‐clockwise with very short travel distance toward the 6 o'clock position then flip the heart to the left to complete the anastomosis in a counter clock fashion up to 12 o'clock position. Check for any loose suture line before tying to complete the anastomosis at 12 o'clock position. A secure anastomosis should be symmetrical and must approximated the donor and recipient intima without gaps. Keep only 5‐7 cm of working suture and truncate the remainder to keep the anastomosis and suture material in the surgical field.
13. Once the aortic anastomosis is done, free the pulmonary artery from the aorta if needed and orient it correctly.
14. Before starting pulmonary artery anastomosis, inject 3mL of subcutaneous (nape of the neck) normal saline in anticipation of a significant drop in afterload once the vascular clamp is released. Also, decrease isoflurane concentration from 3% to 2.5%, then to 2% as the pulmonary artery anastomosis is near completion. This is to prevent respiratory depression and CO_2_ retention. Monitor the respiratory pattern of the rat and the adequate depth of anesthesia.
15. Open the IVC cephalad in relation to the aortic anastomosis with a 30G needle and extend it carefully with micro‐iris scissors. The IVC tears very easily and requires extreme caution when handling. Flush the IVC with 0.2‐0.3 mL of the saline with 100 units of heparin/mL, both cranially and caudally. A small number of blood clots may be seen, and they need to be carefully removed.
16. Unlike the aortic anastomosis start the pulmonary anastomosis without a stay suture. Placing a stay suture at 6 o'clock position at this time significantly obscures the suture line, instead start at 12 o'clock position with needle going from outside to in on donor pulmonary artery and from inside to out on recipient IVC. Tie the suture and first complete the back wall anastomosis in a clockwise fashion. Once at 6 o'clock position, use a separate stitch to place a stay stitch, this will facilitate keeping anastomosis from narrowing and also allows to maintain symmetry. Once the stay stitch is in place, continue suturing clockwise until reaching 12 o'clock position, and then tie it to the short end of suture from the previous tie. Be careful not to cinch the anastomosis because any narrowing limits venous drainage from the heart.
17. Ideally, the two anastomoses should be completed in 30 minutes or less from the aorta/IVC occlusion time (Figure 10).
18. Once the anastomosis is finished, place small pieces of Surgicel^®^ Absorbable Hemostat over the anastomosis for needle hole hemostasis.
19. Unclamp the vessels, and add more Surgicel^®^ over the needle holes as needed. Cover with small surgical gauze. The transplanted heart will start beating with occasional fibrillations before resuming rhythmic breathing. As long as the heart is beating and there is no overt bleeding, leave the surgical gauze in place for 5 minutes.
20. If satisfied with hemostasis, after 5 minutes remove the gauze carefully, irrigate with saline then return the bowel back into the abdominal cavity. Place omentum over the anastomosed to help with hemostasis. The abdominal wall can then be closed in two layers with 5‐0 monocryl on a 13 mm cutting needle by first closing the linea alba, then closing the skin. Place 2% lidocaine ointment over the abdominal wall before closing the skin.

### 2.4 Postoperative care

1. After completing the closure of the abdominal incision, turn the rat back onto the belly.
2. Continue isoflurane via nose cone at a concentration of 1% and provide appropriate pain medications. After 5 minutes, decrease the isoflurane concentration to 0.5% then stop after an additional 5 minutes.
3. If spontaneous breathing is regular, it is safe to move the rat into a clean recovery cage placed on top of a warm pad to complete the recovery process.
4. Adequate pain control is of outmost importance. Sudden movements may result in bleeding risk or a twist in the anastomosis, which may compromise coronary venous drainage and may result in loss of graft function.

With meticulous sterility, cardioprotection of donor heart, close monitoring, and resuscitation of the recipient along with efficient surgical procedure, a heterotopic DCD heart transplantation can be done safely and with high success.

## 3 DISCUSSION

The first successful heterotopic heart transplantation (HTx) in a rat model was described by Abbott et al^3^ in 1964; however, until the modified description of Ono and Lindsey in 1969, the surgical success rate of heart transplantation was very low.^4^ The end‐to‐end anastomosis of donor and recipient aorta was practiced with a very high rate of paraplegia and low recipient survival. The end to side anastomosis, as described by Ono and Lindsey, decreased the risk of paraplegia and resulted in better recipient survival rates. Despite the improvements in anastomotic technique, the need for circumferential dissection of abdominal aorta, inferior venacava and ligation of lumbar vessels resulted in up to 60% of recipients suffering with paraparesis and paraplegia with variable survival.^5^ The technique of HTx we described here in detail, takes into account the improvements noted from the published literature and our observations to establish a successful DCD HTx model in rat. The success of heterotopic heart transplantation in a rodent model depends on several factors including, appropriate choice of anesthesia, hemostatic vascular anastomosis, intraoperative temperature regulation, and close monitoring during recovery (Table 1).

The anesthetic used by the original description of rodent HTx was pentobarbital sodium. Pentobarbital is a potent anesthetic, but its duration of action is variable, requiring repeat administration and potential for cardiotoxicity to the recipient. Lately, its availability also is limited. An alternate anesthetic agent that was commonly used in combination is ketamine plus dexmedetomidine. Ketamine is a potent analgesic, but it has less sedative action requiring dexmedetomidine to supplement as a sedative agent. Experience with this combination resulted in inconsistent duration and depth of anesthesia with poor surgical results.^5^ Isoflurane is a volatile anesthetic with rapid onset, deep anesthesia, and fast recovery. It is easy to administer and has been the choice of anesthetic in rodent surgeries. It is administered at a concentration of 3%‐5% for induction and between 1.5% and 2.5% for maintenance. In our pilot study of rat HTx, we noticed CO_2_ retention with 3% maintenance for the duration of surgery. However, when we decreased the concentration to 1.5%‐2% for the last few minutes of the procedure the CO_2_ retention has not been an issue, and the rats spontaneously resumed unassisted breathing at the end of the procedure. We recommend for DCD rat heart transplantation an induction with 3% isoflurane followed by a maintenance dose of 2.0%‐2.5% until the last 5 minutes, where the isoflurane concentration can be decreased to between 1.5% and 2% depending on the respiratory pattern of the recipient rat.

A DCD donor heart, in general, is stiff and takes up more space in the abdomen of the recipient during anastomosis, thereby adding tension on suture lines, leading to a higher bleeding risk. Due to poor recovery of DCD heart function upon reperfusion, the blood from the coronary sinus pools into the right ventricle and if not propelled forward, will clot and prevent the forward flow of blood in coronaries leading to graft failure. Thus, special attention is required toward the preparation of both the donor heart and recipient in addition to the technique for a successful heterotopic DCD heart transplantation. We described in detail the steps to overcome the limitations set by the DCD heart.

We recommend the duration of global ischemia for DCD hearts to be 25 minutes or less, which correlates with other published reports^9^ and clinical observations.^2^ DCD‐related ischemia is better tolerated in younger rats ages 2‐3 months,^5^ and as such, we recommend limiting the donor rat age to less than 16 weeks, with better results achieved with rats younger than 12 weeks. Without any cardioplegia, a rat DCD heart remains stiff and in asystole upon reperfusion. Delivery of cardioplegia with the cannulation of the common carotid artery and occlusion of the transverse aorta allows for accurate delivery of cardioplegia and better protection of the heart. The cardioplegia setup described above resulted in survival of DCD hearts transplanted from <20% to over 70%.

Microvascular instruments are essential to the efficient conduct of surgery. Blunt dissection with cotton tip dissectors allows for opening in the retroperitoneum and isolation of major abdominal vessels from the surrounding tissue. Most published reports on rat heterotopic heart transplantation utilized microvascular clips to obtain proximal and distal control on abdominal aorta/inferior vena cava.^4^, ^5^, ^9^ Microvascular clip application to isolate the abdominal aorta and vena cava requires separating the infrarenal segments of these vessels and ligating the lumbar branches to prevent back bleeding. This adds valuable time and risk of paraplegia to the recipient. We adopted a partial occlusion clamp technique (Cooley ATRAUMATA‐vascular clamp; 115 mm, 4½, Aesculap, Tuttlingen, Germany) as described by Wang et al.^6^ This clamp provides immediate isolation of infra‐renal abdominal aorta and IVC without the need for dissecting them or separating the lumbar vessels. We were able to complete the vascular anastomosis consistently in 30 minutes or less by utilizing this clamp.

Microvascular instruments with pointed ends are essential for rat heterotopic heart transplantation. We adopted the instrument set as described by MacDonald et al,^10^ which included pointed forceps, pointed end needle driver and angled microvascular scissors. We used 8‐0 monofilament suture for both aortic and pulmonary artery anastomosis. A short tapered needle (4 mm, 3/8 of a circle) is preferred over a longer needle.

Hemostasis is critical to the survival of the recipient, and we advocate avoiding anticoagulant in the recipient. Once clamped, aorta and IVC are opened in the long axis with a 45° angle micro‐scissors. The opening should match the width of the lumen of the donor aorta and pulmonary artery. We recommend starting anastomosis at 12 o'clock position on the recipient aorta in relation to the face of a clock, as seen by the operator. We also advocate placing stay sutures at the 6 o'clock position on the vascular opening to facilitate symmetrical anastomosis. The pulmonary artery anastomosis technique we propose is similar to once described in the literature,^5^, ^6^ where the posterior anastomosis (close to the aortic suture line) is performed first, starting from 12 o'clock position to visualize the sowing margins better. The same 8‐0 monofilament suture is used for anastomosis. The anastomosis width on the IVC should match the width of the pulmonary artery lumen to prevent any resistance to coronary sinus drainage via the right ventricle.

During the pulmonary artery anastomosis, we recommend to decrease the isoflurane concentration to 2% or less in preparation for waking up from anesthesia. In addition, we routinely administer 3 mL of normal saline (0.9% NaCl) subcutaneously over the nape of the neck to account for insensible losses and potential blood loss from the suture line. We recommend administering long‐acting analgesic (eg, buprenorphine) to allow adequate time for release and desired effect. Before releasing the vascular occlusion clamp, we advocate applying small amounts of Surgicel^®^ as a hemostatic agent to prevent needle hole bleeding. We recommend placing the rat in a pre‐heated cage on a warm pad at 37‐38°C to prevent post‐operative hypothermia.

We incorporated most of the advancements that are described in the literature for successful heterotopic rat heart transplantation and added several steps that we learned from our own experience to achieve over 90% survival in recipients following the above detailed techniques. The result is improved success with DCD heterotopic heart transplantation in rats.

## CONFLICT OF INTEREST

The authors have no conflicts of interest to declare that compromise the quality of this article.
